# Induction of oxidative stress as a mechanism of action of chemopreventive agents against cancer

**DOI:** 10.1038/sj.bjc.6604225

**Published:** 2008-02-05

**Authors:** B Rigas, Y Sun

**Affiliations:** 1 Division of Cancer Prevention, Department of Medicine, Stony Brook University, Stony Brook, NY 11794-5200, USA

**Keywords:** redox, oxidative stress, NO-aspirin, chemoprevention

## Abstract

Prevention is a promising option for the control of cancer. Cellular redox changes have emerged as a pivotal and proximal event in cancer. In this review, we provide a brief background on redox biochemistry, discuss the important distinction between redox signalling and oxidative stress, and outline the ‘multiple biological personalities’ of reactive oxygen and nitrogen species: at low concentrations they protect the cell; at higher concentrations they can damage many biological molecules, such as DNA, proteins, and lipids; and, as we argue here, they may also prevent cancer by initiating the death of the transformed cell. Nitric oxide-donating aspirin is discussed as an instructive example: it generates a state of oxidative stress through which it affects several redox-sensitive signalling pathways, leading ultimately to the elimination of the neoplastic cell via apoptosis or necrosis. As additional examples, we discuss the chemopreventive *n*–3 polyunsaturated fatty acids and conventional nonsteroidal anti-inflammatory drugs, which induce cell death through redox changes. We conclude that modulation of redox biochemistry represents a fruitful approach to cancer prevention.

Prevention has emerged as a promising option for the control of cancer, a defining medical challenge of our time. The ultimate aim of prevention is to accomplish one or more of the following three goals: eliminate the causative agent (e.g., smoking cessation); remove a precancerous lesion (e.g., endoscopic resection of colon adenomas); or counteract pathogenetic pathways (e.g., efforts to prevent cancer using COX-2 inhibitors). Some, although slow, progress has been achieved with the first two approaches, but to date mechanism-driven efforts have rendered no major gains.

Work in recent years has generated enhanced appreciation of the complexity and relevance of pathways altered in the cancer cell. Cellular redox changes, much more explored in cardiology than in oncology (Gutierrez *et al*, 2006), emerge as a pivotal and proximal event in cancer. Initially thought of as ‘the bad guy’, mainly because oxidative stress promotes DNA damage, redox changes may mediate cancer prevention by natural and pharmaceutical agents. After providing a brief background to redox biochemistry, we focus on the chemopreventive nitric oxide-donating aspirin (NO-ASA) to illustrate the concept that altering the redox status of the cancer cell represents a valid strategy for cancer prevention.

## REDOX BIOCHEMISTRY: THE BASICS

Redox biochemistry is fundamental to life. The energy needs of complex organisms require vast amounts of ATP. The supply of ATP depends heavily on redox chemistry, as it is driven by changes in free energy associated with electron or hydrogen transfers (Frein *et al*, 2005). Technically, redox, shorthand for reduction/oxidation, describes all chemical reactions in which the oxidation state of atoms changes. In simpler terms, oxidation describes the loss of electrons by a molecule, atom, or ion, with reduction describing the gain of electrons by the same. Redox signalling is the concept that electron-transfer processes play a key messenger role in biological systems. At the heart of redox signalling are the so-called reactive oxygen species (ROS), a term that includes oxygen radicals (e.g., O_2_^•−^ and OH^•^) and also nonradical derivatives of O_2_ (H_2_O_2_). The discovery of the reactive nitrogen species expanded this term to reactive oxygen and nitrogen species (RONS) (Table 1). Free radicals contain one or more unpaired electrons. Since all molecules seek to be balanced, that is, to have an equal number of protons and electrons, the unpaired electron spins of these radicals make them highly reactive.

Reactive oxygen and nitrogen species are produced continuously by the mitochondria (O_2_^•−^, H_2_O_2_, and OH^•^) of most cells and also by cytochrome *P*450 (O_2_^•−^ and H_2_O_2_), macrophages (O_2_^•−^, H_2_O_2_, and NO^•^), and peroxisomes (H_2_O_2_) (Klaunig and Kamendulis, 2004; Genestra, 2007). During mitochondrial oxidative metabolism about 5% of oxygen is converted primarily into O_2_^•−^, whereas 95% of it is reduced to water. Given the high reactivity of RONS, it is not surprising that the cell has invested heavily into an antioxidant defence system to contain RONS. This defence system includes (a) classic antioxidant enzymes, such as superoxide dismutase (SOD), catalase, glutathione (GSH) peroxidase, glutaredoxin, and thioredoxin. These enzymes are distributed in mitochondria, peroxisomes, and cytoplasm. (b) Nonclassic antioxidant enzymes, for example, haem oxygenase-1. (c) Phase II detoxifying enzymes, recently shown to be protective, such as GSH reductase, NQO1, and GSH transferase; and (d) nonenzymatic antioxidants, such as vitamins E and C, GSH, and catechins.

## RONS: THEIR MULTIPLE BIOLOGICAL PERSONALITIES

For years, the intuitive, although perhaps uncritical, assumption has been that all RONS are bad for the cell. The consequent response has been to try to suppress RONS, hoping to prevent or even reverse RONS-related biological damage. Reactive oxygen and nitrogen species, however, have what could be termed ‘multiple biological personalities’: at low concentrations they protect the cell; at higher concentrations they can damage many biological molecules, such as DNA, proteins, and lipids; and yet, as we will argue here, they may also help prevent cancer by initiating the death of the transformed cell.

An important conceptual distinction has become clear in the past several years (even though adequate terminology has not yet been proposed) (Frein *et al*, 2005; Halliwell, 2007). First, there exists a network of redox-based regulatory mechanisms that are often quite relevant to carcinogenesis. Second, disturbed redox equilibrium is pathophysiological, having been described for years as ‘oxidative stress’. These findings have led to the recent delineation of redox signalling and oxidative stress. Redox signalling is a reversible phase of physiological regulatory reactions occurring over shorter time periods; they concern primarily the main cellular redox systems, for example, GSH, ascorbate, vitamin E, lipoic acid, NADPH, or NADH. The oxidative reactions, leading to posttranslational protein modification, are returned to the resting state by reductive pathways. Such modifications include glutathiolation, *S*-nitrosylation, methionine sulphoxidation, and oxidations with disulphide formation. In contrast, oxidative stress denotes a persistent (over longer time periods) and often irreversible oxidative shift that characterises a pathophysiological state. It has been defined as an imbalance between oxidants and antioxidants in favour of the former, resulting in increased cellular levels of RONS. Oxidative stress is implicated in the pathogenesis of several diseases such as cancer, cardiovascular and neurodegenerative disorders, sepsis, reperfusion damage, rheumatoid arthritis, and diabetes.

There is significant, although incomplete, evidence for a role of RONS in cancer, including genotoxicity, promotion of transformed cell growth, and angiogenesis as well as regulation of apoptosis. A ‘persistent oxidative stress in cancer’ has been hypothesised, for example, in Toyokuni *et al* (1995). Such oxidative stress was considered to contribute to oncogene activation, genomic instability, chemotherapy resistance, and even invasion and metastasis. Nuclear factor-*κ*B (NF-*κ*B), MAPK cascades as well as GSH and related antioxidant pathways were hypothesised to be the mediators. Chronic inflammation, widely connected to carcinogenesis, is also a source of RONS and a linkage of inflammation-generated RONS to cancer has also been postulated. The hypoxia-inducible factor-1*α* (HIF-1*α*) is linked to cancer through its regulation by RONS (Pouyssegur and Mechta-Grigoriou, 2006); in particular, RONS signalling may account for the high levels of HIF-1*α* in normoxic areas of tumours. Hypoxia-inducible factor promotes survival in low oxygen conditions, like those encountered in cancer, by upregulating an array of hypoxia-induced genes, including the vascular endothelial factor, which promotes angiogenesis. Finally, RONS have been associated with the induction of apoptotic and necrotic cell death, the specific outcome depending, among others, on the cellular levels of RONS.

## NO-ASA: A CASE OF RONS-MEDIATED CANCER PREVENTION

**Figure 1:**
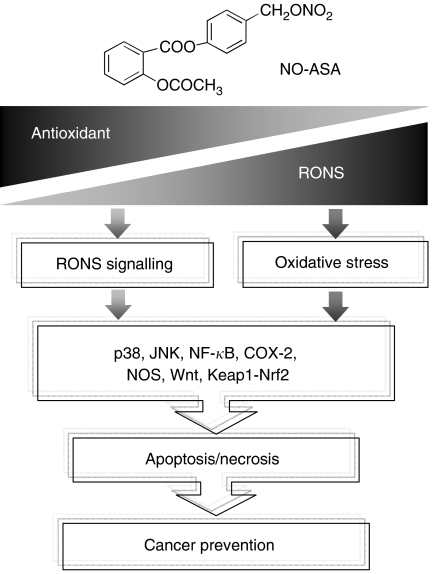
Nitric oxide-donating aspirin, RONS, and cancer prevention. Nitric oxide-donating aspirin induces cell death by altering the redox balance. It achieves it by reducing the levels of cellular antioxidants, such as GSH, and/or by generating RONS, such as superoxide anion, nitric oxide, peroxynitrite, and hydrogen peroxide. The net effect is time and concentration dependently increased RONS levels. Moderately elevated levels of RONS initiate RONS signalling, such as through MAPKs (p38, JNK), NF-*κ*B, COX-2, NOS, Wnt, and Keap1-Nrf2, which can lead to apoptotic cell death. Excessive RONS levels constitute oxidative stress, which drives the cell directly to apoptosis or necrosis. The redox-mediated cytokinetic effect of NO-ASA, which eliminates neoplastic cells, constitutes perhaps its major chemopreventive action against cancer.

Nitric oxide-donating aspirin consists of conventional aspirin to which the NO-releasing moiety –ONO_2_ has been attached via a chemical linker as reviewed in Rigas and Kashfi (2004) (Figure 1). Studies using animal tumour models have established the strong chemopreventive effect of NO-ASA against colon and pancreatic cancer. The remarkably potent inhibition by NO-ASA of the growth of various human cell lines, for example, colon, prostate, lung, pancreas, tonsil, breast cancer, and leukaemia, suggests that a similar chemopreventive effect on these types of malignancies is also possible.

The growth inhibitory effect of NO-ASA is due to a profound cytokinetic effect consisting of reduced cell proliferation, enhanced cell death, and inhibition of cell cycle phase transitions. We have studied signalling pathways that potentially underlie this effect. NO-ASA (a) inhibited the activation of NF-*κ*B in various cancer cell lines and *in vivo* (Williams *et al*, 2003); (b) inhibited the transcriptional activity of *β*-catenin by diminishing its physical association with TCF (Nath *et al*, 2003); (c) increased the phosphorylation of p38 and JNK, representing two out of the three main MAPK cascades; its effect on the ERK1/2 pathway was minimal (Hundley and Rigas, 2006); (d) inhibited both the catalytic activity and expression of iNOS, the inducible isoform of nitric oxide synthase (NOS) (Spiegel *et al*, 2005); (e) had mixed effects on the COX pathway: it induced the expression of COX-2 (no effect on COX-1) in various cell lines, whereas in animals it inhibited COX activity and the levels of various prostaglandins (Williams *et al*, 2003; Rao *et al*, 2006); and (f) induced the translocation of Nrf2 into the nucleus, promoting its dissociation from its cytoplasmic anchor Keap1 (Gao *et al*, 2006).

A shared characteristic of these pathways is that they are redox sensitive or involved in the regulation of the redox status of the cell. This prompted us to examine whether NO-ASA increased ROS levels. Both O_2_^•−^ and a series of RONS collectively assayed by the DCFDA probe were elevated in response to NO-ASA (Gao *et al*, 2005); subsequent work has revealed increased intracellular levels of H_2_O_2_, peroxynitrite, and NO (Sun Y, Rigas B, unpublished data). The oxidative stress that was thus induced led to apoptosis by activating the intrinsic apoptosis pathway. Pretreatment of the cells with *N*-acetylcysteine abrogated cell death and restored cell growth to control levels, clearly indicating that generating a state of oxidative stress in the target cells was a critical proximal event in the action of NO-ASA in cancer. Nitric oxide-donating aspirin also induced apoptosis in small intestinal cells of *Min* mice that bear a somatic mutation predisposing them to intestinal cancer (Williams *et al*, 2004). A derivative of NO-ASA in which the NO-donating moiety was substituted by diethylphosphate also induced oxidative stress (Kashfi and Rigas, 2007; Sun Y, Rigas B, unpublished data). Interestingly, this compound, provisionally termed ‘phosphoaspirin’ is quite effective in preventing colon cancer in a xenograft animal model (Rigas and Kozoni, 2008). This finding emphasises that the oxidative stress induced by NO-ASA is not due exclusively to the NO that it releases.

The specifics of this effect are currently unclear but its mechanistic significance is unmistakable. It is quite likely that many of the changes in the signalling pathways mentioned above are derivative events, generated by redox changes that either lead to reversible redox signalling or irreversible oxidative stress that culminates in cell death and thus elimination of the neoplastic cell (Figure 1).

## ADDITIONAL EXAMPLES OF RONS-MEDIATED CANCER PREVENTION: *N*–3 PUFA AND CONVENTIONAL NSAIDS

Chapkin's team, studying the impact of *n*–3 polyunsaturated fatty acids (PUFA) (dietary fish oil) on colon carcinogenesis, showed that *n*–3 PUFA inhibited carcinogenesis through modulation of ROS levels. Several mechanistic findings supported this notion – and underscored the complexity of the relevant phenomena. Fish oil reduced oxidative DNA damage, lowered cell proliferation, and increased apoptosis in rats, preventing colon cancer (Hong *et al*, 2005). Interestingly, *n*–3 PUFA induced apoptosis either alone or synergistically when combined with butyrate (a product of fermentable fibre). Apoptosis was associated with increased ROS production and inhibition of antioxidant enzymes, such as SOD, catalase, and GSH peroxidase (Sanders *et al*, 2004). Membrane phospholipid hydroperoxides, one of the ROS (Sanders *et al*, 2004; Ng *et al*, 2005), disrupted the mitochondrial permeability transition pore and released cytochrome *c* into cytosol, triggering apoptosis. *n*–3 PUFA induced oxidative damage not only of the mitochondrial membrane but also of other membrane structures, such as lipid rafts in plasma membrane, which play an important role in the modulation and integration of cell signalling (Switzer *et al*, 2004). Reactive oxygen species generation by these chemopreventive interventions was the critical biochemical change that appeared to precede a host of relevant signalling events.

Conventional NSAIDs provide another example of how RONS may mediate cancer prevention. Epidemiological and interventional studies have established that NSAIDs reduce the risk of colon cancer and regress colorectal adenomas in patients with familial adenomatous polyposis (Baron, 2003). Besides their classical inhibitory effect on cyclooxygenase, NSAIDs exert pleiotropic non-COX effects (Hanif *et al*, 1996), including the generation of ROS. Although at times methodologically limited, the relevant literature provides a consensus that treatment of various cancer cell lines with NSAIDs does indeed generate increased ROS levels.

Giardina and colleagues determined the effect of the NSAIDs indomethacin and salicylic acid, and the short-chain fatty acids butyrate and propionate on ROS metabolism in the HT-29 human colorectal carcinoma cell line. All these agents increased cellular peroxide generation. Interestingly, (a) these agents sensitised the normally resistant HT-29 cells to TNF-*α*-induced apoptosis, and (b) arachidonic acid, which also increased ROS, synergised with indomethacin in this reaction (Giardina and Inan, 1998; Giardina *et al*, 1999). The NSAID sulindac, and its derivatives sulindac sulphide and sulindac sulphone increased RONS levels in DLD-1 colon cancer cells, assayed with a probe that reacts with several such species (Oh *et al*, 2003; Adachi *et al*, 2007). Of note, these three compounds had broader effects on redox homeostasis, as they increased transiently the activity of catalase, SOD, and GSH peroxidase. An antioxidant effect was also suggested by *in vitro* studies assessing scavenging activity of sulindac and its derivatives against various RONS (Fernandes *et al*, 2003; Costa *et al*, 2005). It is, however, the balance of these antithetic events that determines the final redox status and all evidence indicates it to be pro-oxidant. A study by Chung *et al* (2003) demonstrated that sodium salicylate mediates ROS production followed by decreased mitochondrial membrane potential, release of cytochrome *c*, and activation of caspase-9 and -3. Reactive oxygen species scavengers and an inhibitor of NADPH oxidase as well as expression of a dominant-negative form of Rac1 blocked ROS production and the consequent apoptotic cell death.

Chemotherapeutic agents are also known to induce the production of RONS. For example, the topoisomerase inhibitor etoposide (Oh *et al*, 2007), arsenic trioxide (Nakagawa *et al*, 2002), and cisplatin (Berndtsson *et al*, 2007) all induced cell death in various cancers through oxidative stress.

## CONCLUSIONS

Our data and those reported by others make it clear that an array of agents controlling cancer either through prevention or treatment have as their common denominator the production of RONS. An additional point is equally clear: a significant fraction of cell signalling pathways modulated by these agents is RONS dependent or RONS responsive; activation of such pathways culminates to the death of the relevant cell through either apoptosis or its variants or through necrosis (Heck *et al*, 2005).

That the chemopreventive agents discussed here work by increasing RONS levels suggests that modulation of redox biochemistry represents a fruitful approach to cancer prevention. Conceptualising this underappreciated target for cancer control should guide efforts to develop novel agents or combinations thereof, in which RONS levels are the guiding read-out. This approach illustrates how mechanistic understanding of the pharmacological mode of action of effective agents can be translated into an effective chemoprevention strategy.

## Figures and Tables

**Table 1:** Reactive oxygen and nitrogen species (RONS)

**Reactive oxygen species**		**Reactive nitrogen species**	
**Radicals**	**Nonradicals**	**Radicals**	**Nonradicals**
Superoxide (O_2_^•−^), hydroxyl (OH^•^), peroxyl (RO_2_^•^), alkoxyl (RO^•^), hydroperoxyl (HO_2_^•^)	Hydrogen peroxide (H_2_O_2_), hypochlorous acid (HOCl), ozone (O_3_), singlet oxygen (^1^ΔgO_2_), peroxynitrite (ONOO^−^)	Nitric oxide (NO^•^), nitrogen dioxide (NO_2_^•^)	Nitrous acid (HNO_2_), dinitrogen trioxide/tetroxide (N_2_O_3_/N_2_O_4_), nitronium (nitryl) ion (NO_2_^+^), peroxynitrite (ONOO^−^), alkyl peroxynitrite (ROONO), nitroxyl anion (NO^−^), nitrosyl cation (NO^+^), nitryl chloride (NO_2_Cl)
